# Steering the reaction pathway of syngas-to-light olefins with coordination unsaturated sites of ZnGaO_x_ spinel

**DOI:** 10.1038/s41467-022-30344-1

**Published:** 2022-05-18

**Authors:** Na Li, Yifeng Zhu, Feng Jiao, Xiulian Pan, Qike Jiang, Jun Cai, Yifan Li, Wei Tong, Changqi Xu, Shengcheng Qu, Bing Bai, Dengyun Miao, Zhi Liu, Xinhe Bao

**Affiliations:** 1 State Key Laboratory of Catalysis, Dalian Institute of Chemical Physics, Chinese Academy of Sciences, 457 Zhongshan Road, Dalian, 116023 PR China; 2 Dalian National Laboratory for Clean Energy, Dalian Institute of Chemical Physics, Chinese Academy of Sciences, 457 Zhongshan Road, Dalian, 116023 PR China; 3 University of Chinese Academy of Sciences, 100049 Beijing, PR China; 4 State Key Laboratory of Functional Materials for Informatics, Shanghai Institute of Microsystem and Information Technology, Chinese Academy of Sciences, Shanghai, 200050 PR China; 5 School of Physical Science and Technology, ShanghaiTech University, Shanghai, 201210 PR China; 6 Department of Chemical Physics, University of Science and Technology of China, Jinzhai Road 96, Hefei, 230026 PR China; 7 High Magnetic Field Laboratory, Hefei Institutes of Physical Science, Chinese Academy of Sciences, Hefei, 230031 PR China

**Keywords:** Catalytic mechanisms, Heterogeneous catalysis, Chemical engineering

## Abstract

Significant progress has been demonstrated in the development of bifunctional oxide-zeolite catalyst concept to tackle the selectivity challenge in syngas chemistry. Despite general recognition on the importance of defect sites of metal oxides for CO/H_2_ activation, the actual structure and catalytic roles are far from being well understood. We demonstrate here that syngas conversion can be steered along a highly active and selective pathway towards light olefins via ketene-acetate (acetyl) intermediates by the surface with coordination unsaturated metal species, oxygen vacancies and zinc vacancies over ZnGaO_x_ spinel−SAPO-34 composites. It gives 75.6% light-olefins selectivity and 49.5% CO conversion. By contrast, spinel−SAPO-34 containing only a small amount of oxygen vacancies and zinc vacancies gives only 14.9% light olefins selectivity at 6.6% CO conversion under the same condition. These findings reveal the importance to tailor the structure of metal oxides with coordination unsaturated metal sites/oxygen vacancies in selectivity control within the oxide-zeolite framework for syngas conversion and being anticipated also for CO_2_ hydrogenation.

## Abstract (web summary)

Great progress has been made in the development of bifunctional oxide-zeolite catalysts to tackle the selectivity challenge in syngas chemistry. Here the authors show syngas conversion can be steered along a highly active and selective pathway towards light olefins via ketene acetate (acetyl) intermediates.

## Introduction

Syngas is an important intermediate platform for the utilization of carbon resources such as coal, natural gas and biomass, which can be converted to a variety of high-value chemicals and fuels. The selectivity control in syngas chemistry remains a challenge although significant progress has been made in fundamental studies and industrial applications of Fischer-Tropsch synthesis (FTS) technology since its invention almost a century ago^1,2^. It was demonstrated that composite catalysts by coupling partially reducible metal oxides and zeolites or zeotypes (OXZEO) enabled syngas direct conversion to a variety of chemicals, e.g., light olefins, gasoline range isoparaffins, benzene-toluene-xylene (BTX), and even oxygenates, with their selectivities all surpassing the Anderson-Schultz-Flory (ASF) distribution limit^3–7^. For example, the selectivity of light olefins among hydrocarbons reached 80% at 17% CO conversion over ZnCrO_x_-SAPO-34 at 400 °C, 2.5 MPa^3^ while 49% CO conversion and 83% selectivity of light olefins over ZnCrO_x_-AIPO-18 at 390 °C, 10 MPa^8^. ZnCrO_x_-mordenite gave 83% ethylene selectivity and 7% CO conversion at 360 °C, 2.5 MPa^6^. Furthermore, similar metal oxide-zeolite systems were also developed for CO_2_ hydrogenation to a variety of chemicals and fuels. For instance, ZnZrO_x_-SAPO-34 for light olefins synthesis^9^, ZnZrO_x_-ZSM-5^10^, and ZnAlO_x_-H-ZSM-5^11^ for aromatics synthesis, and In_2_O_3_-H-ZSM-5 for gasoline-range hydrocarbon synthesis^12^.

It is generally recognized that within the framework of OXZEO catalyst concept, CO/H_2_ activation takes place over metal oxides and C–C coupling over zeolites^3,4^. The partial reducibility of metal oxides was essential in controlling the overall activity of CO conversion. For example, partially reduced MnO_x_ enabled CO dissociation and conversion to surface carbonate and carbon species, which were converted to CO_2_ and hydrocarbons upon H_2_ introduction. In comparison, no carbonate species were detected on unreduced MnO_x_, revealing the pivotal role of surface oxygen vacancies in syngas conversion^13^. Similarly, the reducibility phenomenon was reported for Zn-based catalysts^13–16^. ZnAl_2_O_4_ with a Zn/Al ratio of 1/2 achieved the highest CO or CO_2_ conversion, which was attributed to the largest amount of oxygen vacancies thus promoting CO and CO_2_ activation and conversion^14^. Zn/Cr ratios also affected H_2_ reduction and thus the formation ability of oxygen vacancies, which significantly influenced the activity and selectivity of syngas conversion^15,17^. ZnO-ZrO_2_ aerogel catalyst provided high surface area and large amount of oxygen vacancies, which also played important roles in bifunctional ZnO-ZrO_2_−ZSM-5 catalyst for CO_2_ hydrogenation to aromatics^16^. Despite significant progress, the actual structure of oxygen vacancies, and their catalytic roles are far from being well understood in OXZEO catalysis.

Here, we show that the reaction pathways of syngas conversion strongly depend on the defect sites of metal oxides and hence the final product distribution. Partially reducible ZnGaO_x_ spinel containing coordination unsaturated Ga^3+^ sites and oxygen vacancies steers syngas conversion pathway towards light olefins, whereas spinel with a similar composition but only little oxygen vacancies and zinc vacancies is remarkably less active and non-selective for light olefins.

## Results

### Structure and catalytic performance of ZnGaO_x_−SAPO-34 composites

**Fig. 1:**
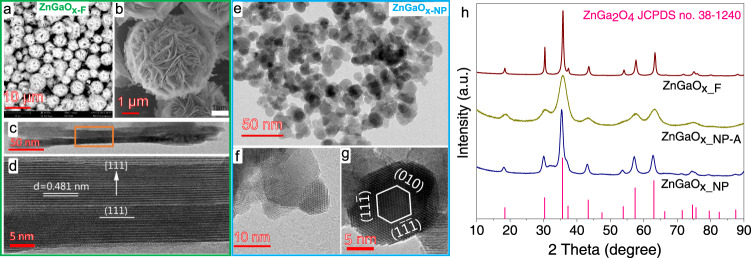
ZnGaO_x_ structure. **a**–**d** ZnGaO_x_F_ sample. **a**, **b** Scanning electron microscopy (SEM) images. **c**, **d** High-resolution transmission electron microscopy (HRTEM) side view images. **d** The enlarged image of a selected area with orange frame in **c** viewed along the [$1\bar{1}0$] orientation. **e**–**g** ZnGaO_x_NP_ sample. TEM images with **g** viewed along the [101] orientation. Note that [uvw] indexed a crystal axis, (hkl) a crystal plane, and {hkl} a group of crystal planes with the same atomic configuration^70^. **h** XRD pattern of ZnGaO_x_ samples.

ZnGaO_x_ oxides prepared by coprecipitation method were denoted as ZnGaO_x_NP_ and those by hydrothermal method were named as ZnGaO_x_F_. The transmission electron microscopy (TEM) and scanning electron microscopy (SEM) images in Fig. 1a–d, and Supplementary Figs. 1, 2a, b, and 3a–d show that the hydrothermal ZnGaO_x_F_ sample exhibits a hydrangea shape formed by thin flakes of ~21 nm thickness and micrometer size on the plane direction. In comparison, all coprecipitation ZnGaO_x_NP_ samples exhibit nanoparticle morphologies (Fig. 1e–g, and Supplementary Figs. 2c, d, 3e–j, and 4), which do not appear to preferentially expose certain crystal faces. Various faces are observed including {111} and {010} in the [101] orientation over ZnGaO_x___NP_ sample (Fig. 1g and Supplementary Fig. 3e–j).

**Fig. 2:**
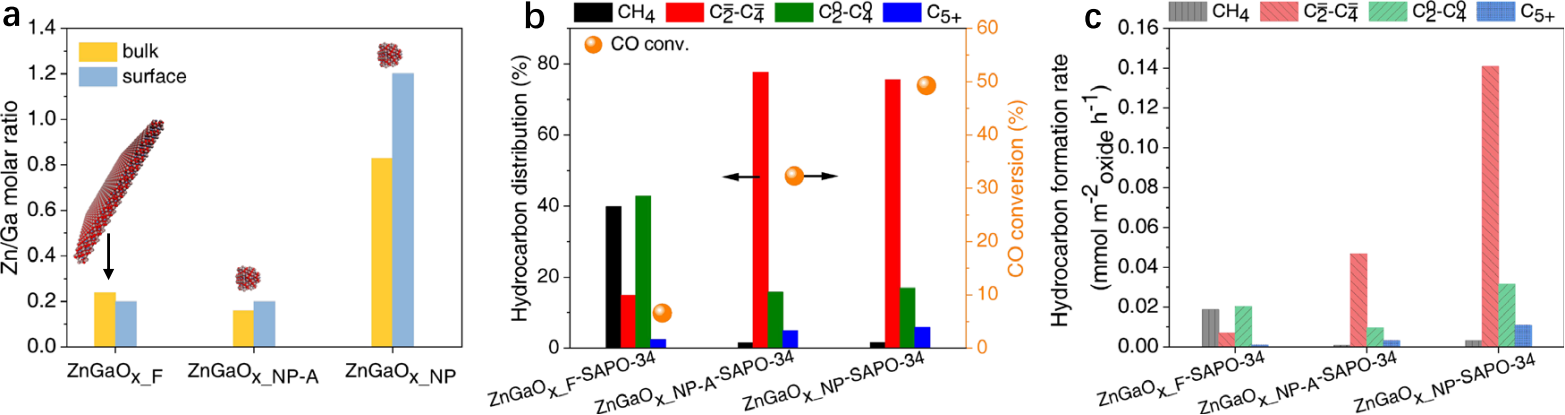
Elemental ratio and reaction performance. **a** Zn/Ga molar ratio of ZnGaO_x_ samples corresponding to the data in Supplementary Table 1. The inset is morphology diagram, with purple, dark pink, and red balls referring to Zn, Ga, and O atoms, respectively. **b** Reaction performance of syngas conversion over ZnGaO_x_−SAPO-34. **c** Hydrocarbon formation rate normalized by specific surface area of oxides. Reaction conditions: OX/ZEO = 1 (mass ratio, 20–40 mesh), H_2_/CO = 2.5 (v/v), 400 °C, 4 MPa, 1600 mL g^−1^ h^−1^.

X-ray diffraction (XRD) patterns confirm that all ZnGaO_x_ samples exhibit the same spinel crystal phase (JCPDS number of 38-1240, Fig. 1h, and Supplementary Figs. 5 and 6a) with the Zn/Ga ratio ranging from 0.2 to 3.1 (Supplementary Table 1). Therefore, they are non-stoichiometric spinels^18,19^. We first compared a nanoparticle spinel ZnGaO_x_NP-A_ with a nanoflake spinel ZnGaO_x_F_ with similar surface and bulk Zn/Ga molar ratios, as analyzed by inductively coupled plasma optical emission spectrometer (ICP-OES), scanning electron microscopy with energy dispersive X-ray detector (SEM-EDX), and X-ray photoelectron spectroscopy (XPS) (Fig. 2a, Supplementary Table 1). Interestingly, these two oxides give significantly different catalytic performance in syngas conversion upon being physically mixed with SAPO-34, respectively (Fig. 2b, Supplementary Table 2). CO conversion over ZnGaO_x_NP-A_−SAPO-34 is 32.3%, almost 5 times higher than 6.6% over ZnGaO_x_F_−SAPO-34, while the light olefins selectivity over the former is also 5 times higher than that over the latter (77.6% versus 14.9%). Note that hydrocarbons selectivity in this study is reported excluding CO_2_ to simplify the discussion since CO_2_ selectivity is similar for all catalysts (Supplementary Table 2). Even being normalized by the specific surface area of oxides, ZnGaO_x_NP-A_ still exhibits a yield of light olefins 7 times higher than ZnGaO_x_F_ (0.047 versus 0.007 mmol m^−2^ h^−1^) (Fig. 2c).

The catalytic activity can be further optimized by varying the composition of ZnGaO_x_, as shown in Supplementary Tables 1 and 2. Notably, all nanoparticle ZnGaO_x_NP_ samples demonstrate much superior performance than ZnGaO_x_F_ upon being physically mixed with SAPO-34 (Fig. 2b, c and Supplementary Table 2). ZnGaO_x_NP_ with a surface Zn/Ga molar ratio of 1.2 (Fig. 2a) gives a highest CO conversion, i.e., 49.3% with 75.6% selectivity of light olefins (Fig. 2b and Supplementary Table 2). This CO conversion is higher than most results reported for Cr-free oxides under similar reaction conditions^20^. The formation activity of light olefins is 0.14 mmol m^−2^ h^−1^, which is 20 times higher than that over ZnGaO_x_F_ (Fig. 2c). Moreover, ZnGaO_x_NP_−SAPO-34 delivers a rather good stability. CO conversion remains at 46% and selectivity of light olefins at 70% after 120 h on stream (Supplementary Fig. 7).

To understand the distinctively different activity of nanoparticle and nanoflake ZnGaO_x_ spinel, we first looked into the activity of ZnGaO_x_NP_ and ZnGaO_x_F_ alone in syngas conversion. The results in Supplementary Table 3 show that both oxides give similar CO conversion around 5.5%. However, they behave significantly different when they combined with SAPO-34 respectively as composites. ZnGaO_x_NP_−SAPO-34 provides CO conversion > 37% and light olefins selectivity > 71% at different OX/ZEO mass ratios (Supplementary Fig. 8a). It indicates that the reaction equilibrium is successfully shifted and the reaction channel from intermediates to light olefins is opened up in the presence of SAPO-34, consistent with a recent theoretical study^21^. Thus, it forms a tandem catalytic process. Furthermore, the composite with OX/ZEO = 1 (mass ratio) gives optimal performance. By contrast, introducing SAPO-34 to ZnGaO_x_F_ hardly affects the overall conversion (<8%) and light olefins selectivity (<16%) with the OX/ZEO ratio changing from 1/2 to 2/1 (Supplementary Fig. 8b, c). The main products are methane (45%) and light paraffins (40%). It implies that ZnGaO_x_F_ is intrinsically of low activity or the intermediates generated over ZnGaO_x_F_ cannot be effectively converted to desired products by SAPO-34 catalyst in contrast to ZnGaO_x_NP_. The reaction most likely has gone through different pathways over the two types of oxides.

### In-situ FT-IR analysis of reaction intermediates over ZnGaO_x_ spinel

**Fig. 3:**
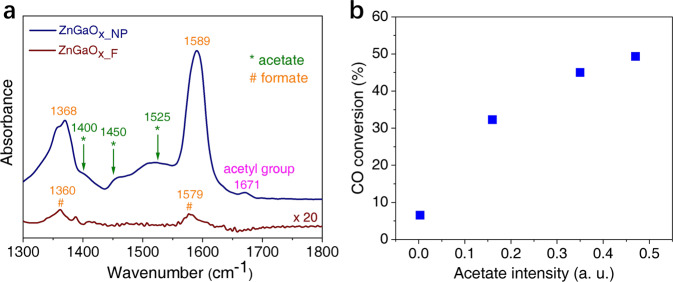
Surface intermediates over ZnGaO_x_ oxides and the relationship with catalytic performance. **a** In-situ FT-IR differential spectra of syngas conversion over H_2_-reduced ZnGaO_x_NP_ (navy line) and ZnGaO_x_F_ (brown line) at 400 °C. **b** CO conversion as a function of acetate intensity at 1525 cm^−1^ of FT-IR spectra of different ZnGaO_x_ samples in Supplementary Fig. 9a.

The in-situ Fourier Transform Infrared (FT-IR) spectra in Fig. 3a confirm the distinct intermediates generated over the two ZnGaO_x_ oxides. Upon exposing ZnGaO_x_NP_ to syngas at 400 °C, two strong absorption bands appear at ~1368 and ~1589 cm^−1^, which are generally ascribed to formate species^22^. Such formate species is also observed over ZnGaO_x_F_, although the intensity is weaker. Formate species has been widely observed over methanol synthesis catalysts and is generally considered to be the precursor of methanol^4,9,23,24^. It was also reported for the metal oxides of the OXZEO composites which go via methanol-olefins pathway^4,8,25,26^. However, neither the intensity of IR formate signal correlates well with CO conversion of the corresponding OXZEO catalysts (Supplementary Fig. 9) nor the methanol concentration produced by ZnGaO_x_ correlates with the hydrocarbons produced by OXZEO catalysts (Supplementary Fig. 10).

In comparison, over ZnGaO_x_NP_ surface, additional signals at ~1400 (*δ*_CH3_), ~1450 (υ_C-O_) and ~1525 cm^−1^ (*υ*_C=O_) are obviously observed (Fig. 3a and Supplementary Fig. 9a), which are characteristic acetate species^27–30^. The band at 1671 cm^−1^ is assigned to acetyl group^27–30^. The acetate species likely originates from ketene being chemisorbed on the surface hydroxyl group, and acetyl group could be the product of hydrogenated ketene in the absence of zeotypes. Acetate species, a product of C–O breaking and C–C coupling, was not reported over typical methanol synthesis catalysts previously^4,9,23,24^. Formation of acetate species over ZnGaO_x-NP_ was also validated by solid-state Nuclear Magnetic Resonance study^31^. Figure 3b displays that CO conversion of ZnGaO_x_−SAPO-34 bifunctional catalysts correlates well with the intensity of the representative IR signal for acetate species (1525 cm^−1^). Therefore, the reaction likely proceeds through ketene/acetyl/acetate pathway over ZnGaO_x_-SAPO-34 although methanol contribution cannot be completely excluded because SAPO-34 is a classical catalyst for methanol-to-olefins.

### Unraveling the coordination unsaturated metal sites over ZnGaO_x_ spinel

**Fig. 4:**
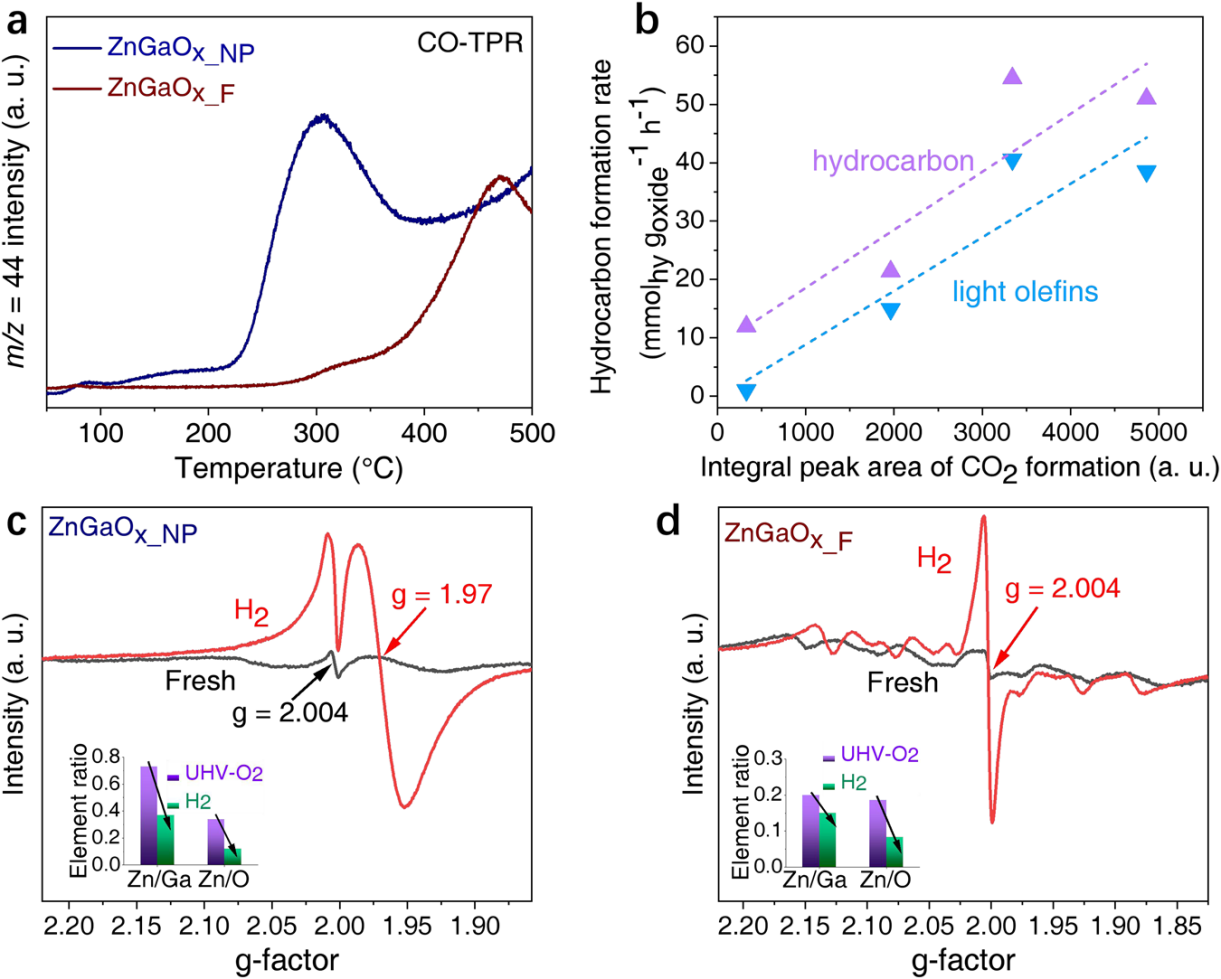
Structural analysis of ZnGaO_x_ by TPR, EPR. **a** CO-TPR profiles with *m/z* = 44 (CO_2_) signals in the effluents monitored by an online mass spectrometer. **b** Mass specific hydrocarbon formation rate as a function of integral area of CO_2_ signals below 400 °C in CO-TPR profiles of different ZnGaO_x_. Reaction conditions: OX/ZEO = 1/4 (mass ratio), H_2_/CO = 2.5 (v/v), 400 °C, 4 MPa, and 20,000 mL g^−1^ h^−1^. **c**, **d** *Quasi-*in-situ EPR spectra of ZnGaO_x_ before and after H_2_ reduction, and the inset showing the surface Zn/Ga and Zn/O ratios determined by AP-XPS results. The purple and green colors refer to treatment conditions of UHV-O_2_ (degassed in ultra-high vacuum, and then exposed to O_2_) and H_2_, respectively. **c** ZnGaO_x_NP_. **d** ZnGaO_x_F_.

To understand the origin of different reaction pathways over the two types of ZnGaO_x_ spinel, we set out to investigate their structures and active sites. Although metal oxides are less studied as catalysts directly, the increasing number of studies have proposed the important role of oxygen vacancies^3,25,26^ in OXZEO catalyzed syngas conversion. Therefore, we turned to CO-temperature programmed reduction (TPR) first. The profiles in Fig. 4a demonstrate a much more facile reduction of ZnGaO_x_NP_ than ZnGaO_x_F_. A strong signal of CO_2_ centers around 300 °C over ZnGaO_x_NP_. This signal appears to overlap with another one starting from ~ 400 °C, which is likely CO disproportionation. Nevertheless, the integrated area of CO_2_ signals below 400 °C in CO-TPR (Fig. 4a, Supplementary Fig. 11, and Supplementary Tables 4 and 5) can still reflect the reducibility. In contrast, the reduction peak is significantly weaker over ZnGaO_x_F_ indicating very few reducible defect sites below 400 °C. H_2_-TPR shows a similar trend (Supplementary Fig. 12). Figure 4b displays that the specific formation rates of hydrocarbons and light olefins correlate monotonically with the reducibility of ZnGaO_x_ oxides regardless of nanoparticles or nanoflakes, which is consistent with previous studies^3,13,25,26^, revealing again the essential role of reducibility.

The reduction process can remove surface oxygen atoms, thereby leaving oxygen vacancies and coordinatively unsaturated metal sites, which are further investigated by *quasi-*in-situ electron paramagnetic resonance (EPR)^32–35^. Figure 4c shows that fresh ZnGaO_x_NP_ exhibits a very weak EPR signal at g = 2.004. However, it intensifies significantly together with a new signal showing up at g = 1.97 upon H_2_ reduction (Supplementary Fig. 13a). In comparison, only a weak signal attributed to manganese impurity^36^ is detected for fresh ZnGaO_x_F_ (Fig. 4d, and Supplementary Fig. 13b), which may has been brought in during catalyst synthesis (Supplementary Table 1). Upon H_2_ reduction, a signal at g = 2.004 appears and no other signals are observed over nanoflakes. g = 2.004 signal was frequently reported to be related to the presence of a free electron in the conduction band^37,38^, or defect sites over singly ionized zinc vacancy of zinc-based oxides^32,39–42^. However, it was also assigned to unpaired electron trapped at an oxygen vacancy site of oxides such as ZnO^35,41,43^, Zn_x_Ga_y_O_z_^37,44,45^, or TiO_2_^38,46^. The assignment of g = 1.97 also remains controversial in different studies, e.g., singly ionized oxygen vacancies with one trapped electron^33,47^, zinc vacancies^42,48,49^, or donor centers such as ionized impurity atoms in the crystal lattice of ZnO oxide^50,51^. Volatility of zinc species has been frequently reported for ZnO and Zn-based oxides, which is facilitated in vacuum and hydrogen atmosphere^52–55^. Thus formation of zinc vacancies over both reduced ZnGaO_x_ can be expected upon H_2_ reduction due to removal of oxygen atoms. This is confirmed by in-situ ambient pressure X-ray photoelectron spectroscopy (AP-XPS) experiments (the insets of Fig. 4c, d and Supplementary Table 6). Therefore, it is reasonable to attribute the g = 2.004 signal in both H_2_-reduced oxides to singly ionized zinc vacancies. Since the reducibility of ZnGaO_x_F_ at 400 °C is relatively low (Fig. 4a and Supplementary Fig. 12), the signal intensity of oxygen vacancies over ZnGaO_x_F_ should also be low, in contrast to significant reduction signal over ZnGaO_x_NP_. Therefore, the g = 1.97 signal of H_2_-reduced ZnGaO_x_NP_ can be attributed to singly ionized oxygen vacancies.

**Fig. 5:**
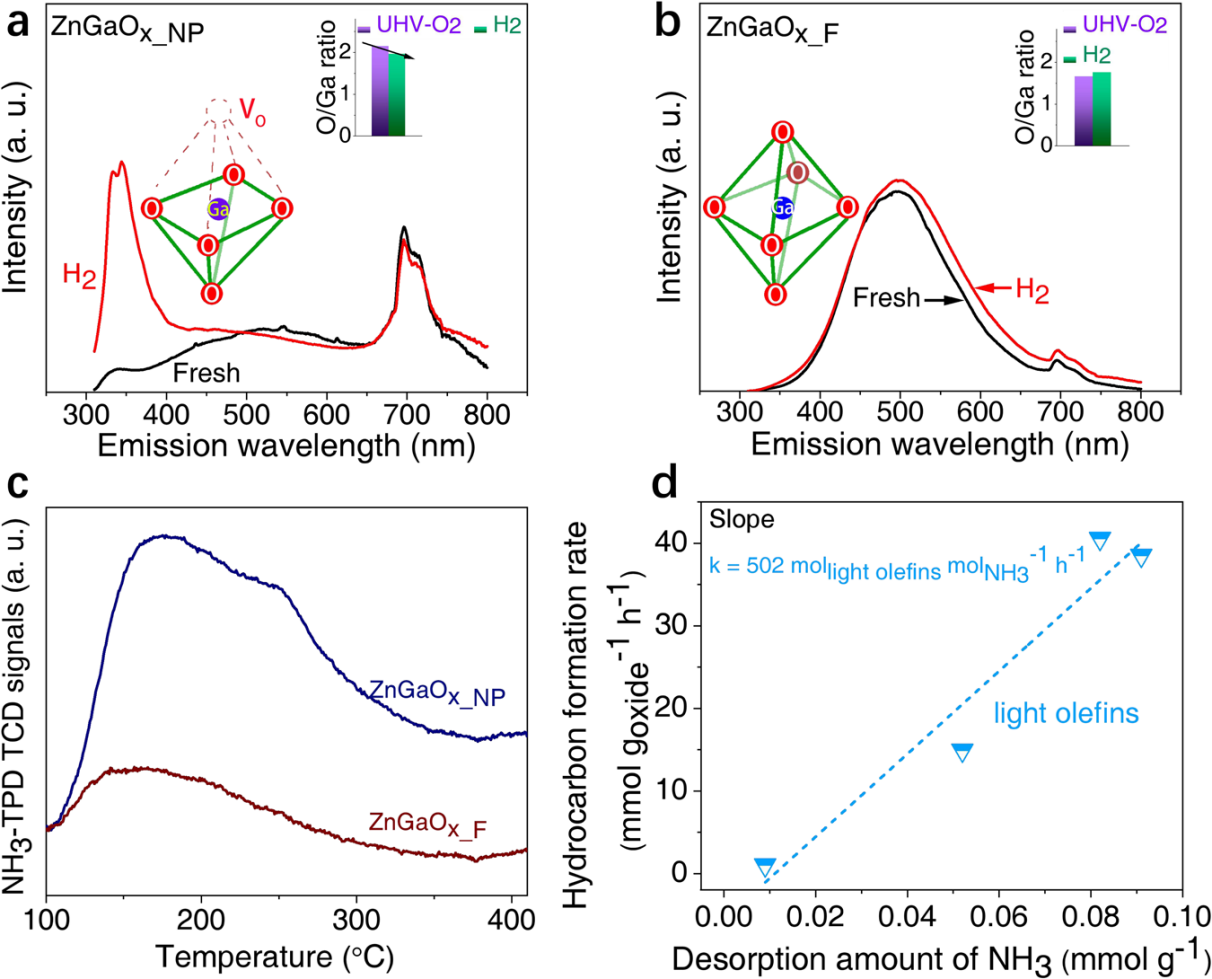
Structural analysis of ZnGaO_x_ by photoluminescence and NH_3_-TPD. **a**, **b** *Quasi-*in-situ Photoluminescence emission spectroscopy. H_2_-reduced samples in comparison to the fresh ones at the excitation wavelength of 290 nm (Supplementary Fig. 14), with the inset showing the surface ratio of O/Ga measured by in-situ AP-XPS. The inset model showing the Ga-O octahedral structure after H_2_ treatment. **a** ZnGaO_x_NP_. **b** ZnGaO_x_F_. **c** NH_3_-TPD profiles of H_2_-reduced ZnGaO_x_ oxides. **d** Mass specific hydrocarbon formation rate as a function of the amount of medium strength acid sites of ZnGaO_x_ estimated by NH_3_-TPD. Reaction conditions: OX/ZEO = 1/4 (mass ratio), H_2_/CO = 2.5 (v/v), 400 °C, 4 MPa, 20,000 mL g^−1^ h^−1^.

Photoluminescence (PL) spectroscopy was conducted to further elucidate the defect structures of ZnGaO_x_ oxides. Figure 5a shows emission peaks around 700 nm over both the fresh and reduced ZnGaO_x_NP_, which are generally attributed to oxygen vacancies^56,57^, in agreement with CO-TPR and EPR results. In addition, upon H_2_ reduction, another emission signal near 350 nm in the ultraviolet (UV) region becomes significantly intensified, consistent with the previous observation for ZnGa_2_O_4_^58^. This was attributed to the formation of the distorted Ga-O octahedral structure, due to the removal of O atoms and thus forming coordination unsaturated Ga^3+^ sites, as displayed in the structure model in Fig. 5a^56,59–61^. This is further validated by a decreased O/Ga ratio of reduced ZnGaO_x-NP_ by AP-XPS (the inset of Fig. 5a), whereas ZnGaO_x_F_ does not show much change of the surface O/Ga ratio (the inset of Fig. 5b). In comparison, the fresh ZnGaO_x_F_ exhibits an emission spectrum significantly different from that of ZnGaO_x_NP_, with a strong and wide signal at 400~650 nm and a weak signal around 700 nm (Fig. 5b). The former signal generally corresponds to the characteristic coordination saturated Ga^3+^ in the Ga-O octahedral structure (model of Fig. 5b)^61,62^ due to the charge transfer between Ga^3+^ ions located at the center of octahedral sites and its six first-neighbor O^2-^ ions^56,61^. Furthermore, the *quasi-*in-situ H_2_-treated ZnGaO_x_F_ at 400 °C gives almost the identical spectrum as the fresh sample, indicating no obvious electronic structure change (Fig. 5b).

**Fig. 6:**
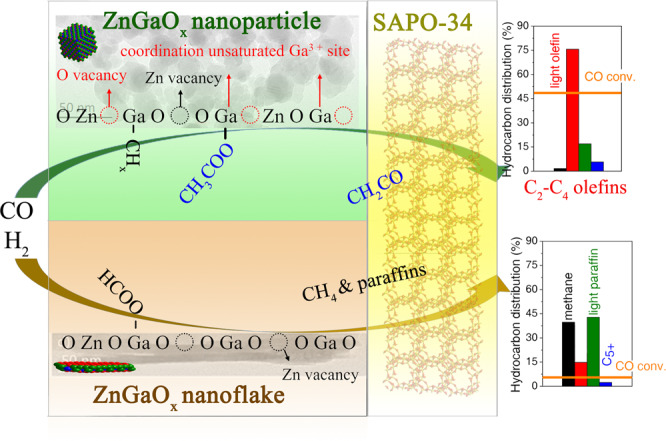
Reaction pathways over ZnGaO_x_-SAPO-34 composites. ZnGaO_x_ oxides containing coordination unsaturated Ga^3+^, oxygen vacancy and zinc vacancy sites are much more active and selective in syngas conversion to light olefins.

The above results indicate that the coordination unsaturated Ga^3+^ sites, oxygen vacancies and zinc vacancies co-exist on reduced ZnGaO_x_NP_ oxide, while only oxygen vacancies and zinc vacancies exist on reduced ZnGaO_x_F_. The coordinatively unsaturated Ga^3+^ sites generally exhibit Lewis acidity^63,64^, which is evidenced by in-situ FT-IR differential spectra of pyridine adsorption on the surface of ZnGaO_x_ oxides (Supplementary Fig. 15). The amount of Lewis acid sites can be quantified by temperature-programmed desorption of ammonia (NH_3_-TPD)^26,65^. Figure 5c and Supplementary Fig. 16a show that all reduced ZnGaO_x_ samples give broad and asymmetric NH_3_ desorption peaks in the range of 100~400 °C, but the concentration differs (Supplementary Table 7). Fitting of NH_3_-TPD profiles (Supplementary Fig. 16b–e and Supplementary Table 8) indicates the presence of weak (around 170 °C) and medium strength acid sites (around 250 °C)^66^. The NH_3_-desorption peak below 200 °C is generally related to hydrogen-bonded physisorption sites^67^, while the peak around 250 °C could be contributed by the defect sites of coordination unsaturated sites^26,65^. Therefore, the number of defect sites on ZnGaO_x_ surface could be quantified by the amount of NH_3_ desorbing from the medium strength acid sites. Interestingly, as shown in Fig. 5d, the mass specific light olefins formation rate is positively correlated with the concentration of these coordination unsaturated metal sites, but not with that of weak strength acid sites (Supplementary Fig. 17). Thus, the light olefins formation rate per defect site can be estimated to be 502 h^−1^, assuming one defect site adsorbing one NH_3_ molecule. The above results demonstrate that the presence of coordination unsaturated Ga^3+^ together with oxygen vacancies and zinc vacancies lead to a much more active ZnGaO_x_ spinel in generating ketene-acetate (acetyl) intermediates. Interestingly, Lai et al. recently also revealed the essential role of coordination unsaturated Cr^3+^ together with the oxygen vacancies in the cleavage of the C–O bond over the highly reduced ZnCr_2_O_4_ (110) surface^21^. Thus, incorporation of SAPO-34 would direct the reaction pathway towards light olefins (Fig. 6).

## Discussion

ZnGaO_x_ spinels with similar compositions but different morphologies were synthesized, which allows to elucidate the catalytic role of different defect sites over metal oxides in OXZEO catalyzed syngas conversion. *Quasi-*in-situ PL, EPR and in-situ FT-IR reveal that ZnGaO_x_NP_ (nanoparticles) is much more reducible, which gives the coordination unsaturated Ga^3+^ species, oxygen vacancies and zinc vacancies. Such a surface facilitates the formation of ketene-acetate (acetyl) intermediates during CO/H_2_ activation, which allows subsequent conversion to light olefins by SAPO-34 and displacement of the reaction equilibrium. Consequently, the selectivity of light olefins reaches 75.6% at 49.3% CO conversion, which is 9 times higher than that obtained by ZnGaO_x_NP_ alone. In comparison, ZnGaO_x_F_ (nanoflakes) containing only oxygen and zinc vacancies catalyzes CO/H_2_ activation generating formate species, which are hardly converted to light olefins by SAPO-34 because CO conversion is only 6.6% and light olefins selectivity is only 14.9%. Instead, the products are mainly composed of CH_4_ and paraffins for both ZnGaO_x_F_ alone and ZnGaO_x_F_−SAPO-34 composite. Although the detailed structure of the active sites and the elementary steps forming intermediates still need further investigation, the results here have already demonstrated that the structure of reducible metal oxides can be tailored to convert syngas selectively to value-added chemicals. These findings are also expected to be applicable to CO_2_ hydrogenation to value-added chemicals and fuels.

## Methods

### Catalyst preparation

ZnGaO_x_NP_, where NP denoted as nanoparticles, was synthesized by a coprecipitation method with the temperature of water bath kept at 60~65 °C and pH kept at 9~10. Aqueous solutions of Zn(NO_3_)_2_·6H_2_O and Ga(NO_3_)_3_ ∙ nH_2_O were prepared as the precursors and their molar ratios were 1:10, 1:6, 1:4, and 1:2, respectively. An aqueous solution of NaOH and Na_2_CO_3_ with a molar ratio of 7.1:1 was used as the precipitant. After precipitation, the suspensions were aged for 2 h under continuous stirring. The precipitates were washed with water, dried at 60 °C and then 110 °C overnight, followed by calcination at 500 °C for 1 h in air, respectively. The resulting samples were named as ZnGaO_x_NP-A_, ZnGaO_x_NP-B_, ZnGaO_x_NP-C_, and ZnGaO_x_NP_, respectively. The effect of calcination temperature, *T*, was also studied in the range of 600~800 °C for ZnGaO_x_NP_ (named as ZnGaO_x_NPT_).

ZnGaO_x_F_, where F denoted as nanoflakes, was prepared by a hydrothermal method by adapting the previously reported method^68^. In detail, 1.21 g 2H_2_O ∙ Zn(CH_3_COO)_2_ and 2.81 g nH_2_O ∙ Ga(NO_3_)_3_ were dissolved in a mixed solution of 110 mL water and 55 mL ethylenediamine. After continuous stirring at room temperature for 1 h, the mixed solution was transferred into a 200 mL hydrothermal kettle, and heated at 180 °C for 24 h. The product was separated by centrifugation, washed several times with water, and then dried overnight at 60 °C, followed by calcination at 500 °C for 1 h in air.

SAPO-34 was synthesized following a hydrothermal method similar to a previous report^3^. Typically, 30% silica sol, AlOOH, 85% phosphoric acid and triethylamine (TEA) were well dispersed in distilled water with a mass ratio of SiO_2_:Al_2_O_3_:H_3_PO_4_:TEA:H_2_O = 0.11:1:1.8:3.4:10. Then the mixture was placed in a Teflon-lined autoclave, and kept at 200 °C for 24 h. The resulting solid product was collected by centrifuging and washed with water till the pH of the supernate was 7.0–7.5. After drying for over 12 h at 110 °C, the white powder was calcined at 550 °C for 4 h in air with a heating rate of 1 °C/min.

### Catalyst characterization

X-ray diffraction (XRD) was measured on a PANalytical Empyrean-100 equipped with a Cu K_α_ radiation source (λ = 1.5418 Å), operated at 40 mA and 40 kV. XRD patterns were recorded in the range of 2 theta = 10~90^o^. The crystal size was estimated using the Scherrer equation. Nitrogen adsorption−desorption experiments were carried out on a Quantachrome NOVA 4200e instrument. Before analysis, samples were degassed under vacuum at 300 °C for 5 h. Isotherms were recorded at 77 K. A non-local density function theory (NLDFT) pore size method was used. High-resolution transmission electron microscopy (HRTEM) images were obtained using a JEOL JEM-2100 electron microscope operated at an accelerating voltage of 200 kV. Before tests, the samples were ultrasonically dispersed in ethanol and a drop of the solution was placed onto a copper grid coated with a thin microgrids support film. High-resolution scanning electron microscopy (HRSEM) images were obtained using a Carl Zeiss Orion NanoFab Helium ion microscope. The low-resolution scanning electron microscopy (SEM) tests were performed on a Phenom proX apparatus with energy dispersive X-ray detector (EDX) elemental analysis. The accelerating voltage was 15 kV. The elemental content was measured using Inductively Coupled Plasma Optical Emission Spectrometer (ICP-OES). Samples were dissolved in aqua regia solution and then sealed in an autoclave with Teflon lining. The autoclave was then placed in a microwave reactor for 0.5 h. The samples were then measured on a PerkinElmer ICP-OES 7300DV apparatus. X-ray photoelectron spectroscopy (XPS) spectra were recorded on a SPECS PHOIBOS-100 spectrometer using an Al K_α_ (hν = 1486.6 eV, 1 eV = 1.603 × 10^−19^ J) X-ray source. The Ga 2*p*_3/2_ binding energy at 1118.7 eV was used for calibration. Typically, only the Ga 2*p*_3/2_ component of the Ga 2*p* and Zn 2*p*_3/2_ of the Zn 2*p* regions are fitted and quantified. The atomic ratio of elements *i* to *j* (${n}_{i}/{n}_{j}$) on the oxide surface was calculated based on

1 $$\frac{{n}_{i}}{{n}_{j}}=\frac{{I}_{i}}{{S}_{i}}\div\frac{{I}_{j}}{{S}_{j}}$$

Where *I* represented the area of the characteristic peak and *S* represented the atomic sensitivity factor in Eq. (1), which was referred to the previous literature^69^. Ambient Pressure X-ray Photoelectron Spectroscopy (AP-XPS) experiments were carried out on SPECS PHOIBOS-150 ambient pressure XPS with Al K_α_ as the X-ray source. ZnGaO_x_ oxides were first degassed in ultra-high vacuum (UHV) at 400 °C, and then heated in 0.5 mbar O_2_. After pretreatment, 0.5 mbar H_2_ was introduced into the analysis chamber. The Ga 2*p*_3/2_ binding energy at 1118.7 eV was used for calibration. Temperature-programmed desorption of NH_3_ (NH_3_‒TPD) was performed on a Micromeritics AutoChem 2910 instrument equipped with a thermal conductivity detector (TCD). Typically, 100 mg sample was loaded into a U-shape reactor. Before NH_3_‒TPD experiment, sample was pretreated at 400 °C for 1 h under flowing H_2_ and then heated under flowing Ar at 500 °C for 1.5 h. After cooling down to 100 °C under flowing Ar, the sample was exposed to 5 vol.% NH_3_/He. Then, the sample was swept by Ar at the same temperature until a stable baseline was obtained. Subsequently, the signal was recorded while the temperature increased from 100 to 600 °C at a heating rate of 10 °C/min. Temperature-programmed reduction (TPR) was performed on another Micromeritics AutoChem 2910 instrument equipped with a TCD. Typically, 100 mg sample was loaded into a U-shape reactor. Before CO-TPR or H_2_-TPR experiment, sample was pretreated at 500 °C for 1 h in flowing Ar. After cooling down to room temperature under flowing Ar, the TPR profile was recorded in 5 vol.% CO/He or 1 vol.% H_2_/Ar at a heating rate of 10 °C/min with the effluents monitored by an online quadrupole mass spectrometer (MS). Electron paramagnetic resonance (EPR) spectra were collected at 7 K on a Bruker A200 EPR spectrometer operated at the X-band frequency using power 1.0 mW, modulation amplitude 4.00 G and receiver gain 10000. The photoluminescence (PL) spectra were measured using QM400 with a Xe-lamp as the excitation source at room temperature. The excitation wavelength was fixed at 290 nm. H_2_ reduction was conducted at 400 °C for 1 h, and then sealed for *quasi-*in-situ study of EPR and PL. In-situ Fourier Transform Infrared (FT-IR) transmission spectra were recorded on a BRUKER INVENIO S spectrometer equipped with a quatz cell. Before tests, sample was pretreated in H_2_ atmosphere at 450 °C for 1 h. After cooling down to room temperature, the background spectrum was recorded. Then, sample was exposed to syngas atmosphere at 1 atm and heated to 400 °C for 1 h. After cooling down to room tempetature, sample spectrum was recorded. Each spectrum was obtained by averaging 32 scans collected at 4 cm^−1^ resolution. The sample spectrum was subtracted by the background spectrum. Pyridine adsorption test was conducted using the same facilities. The sample was degassed in vacuum at 450 °C for 1 h. Then pyridine was introduced at room temperature and subsequently degassed by evacuation. All spectra were collected under room temperature.

### Catalytic reaction tests

Catalytic reaction was performed in a continuous flow, fixed-bed stainless steel reactor equipped with a quartz lining. Typically, 300 mg composite catalyst (20–40 meshes) with oxide/zeolite = 1/1 (mass ratio) was used. 5 vol.% Ar was added to syngas as the internal standard for online gas chromatography (GC) analysis. Reaction was carried out under conditions: H_2_/CO = 2.5 (v/v), 400 °C, 4.0 MPa, gas hourly space velocity (GHSV) = 1600 mL g^−1^ h^−1^ unless otherwise stated. Products were analyzed by an online GC (Agilent 7890B), equipped with a TCD and a flame ionization detector (FID). Hayesep Q and 5 A molecular sieves packed columns were connected to TCD whereas HP-FFAP and HP-AL/S capillary columns were connected to FID. Oxygenates and hydrocarbons up to C_12_ were analyzed by FID, while CO, CO_2_, CH_4_, C_2_H_4_, and C_2_H_6_ were analyzed by TCD. CH_4_, C_2_H_4_, and C_2_H_6_ were taken as a reference bridge between FID and TCD.

CO conversion (${{{\mbox{Conv}}}}_{{{\mbox{CO}}}}$) was calculated on a carbon atom basis, i.e.

2 $${{{{{{\rm{Conv}}}}}}}_{{{{{{\rm{CO}}}}}}}=\frac{{{{{{{\rm{CO}}}}}}}_{{{{{{\rm{inlet}}}}}}}-{{{{{{\rm{CO}}}}}}}_{{{{{{\rm{outlet}}}}}}}}{{{{{{{\rm{CO}}}}}}}_{{{{{{\rm{inlet}}}}}}}}\times 100 \%$$

where ${{{\mbox{CO}}}}_{{{\mbox{inlet}}}}$ and ${{{\mbox{CO}}}}_{{{\mbox{outlet}}}}$ in Eq. (2) represented moles of CO at the inlet and outlet, respectively.

CO_2_ selectivity (${{{\mbox{Sel}}}}_{{{{\mbox{CO}}}}_{2}}$) was calculated according to

3 $${{{{{{\rm{Sel}}}}}}}_{{{{{{{\rm{CO}}}}}}}_{2}}=\frac{{{{{{{\rm{CO}}}}}}}_{{2}_{{{{{{\rm{outlet}}}}}}}}}{{{{{{{\rm{CO}}}}}}}_{{{{{{\rm{inlet}}}}}}}-{{{{{{\rm{CO}}}}}}}_{{{{{{\rm{outlet}}}}}}}}\times 100 \%$$

where ${{{{\mbox{CO}}}}_{2}}_{{{\mbox{outlet}}}}$ in Eq. (3) denoted moles of CO_2_ at the outlet.

The selectivity of individual hydrocarbon C_n_H_m_ (${{{\mbox{Sel}}}}_{{{{\mbox{C}}}}_{{{\mbox{n}}}}{{{\mbox{H}}}}_{{{\mbox{m}}}}}$) among hydrocarbons (free of CO_2_) in Eq. (4) was calculated according to

4 $${{{{{{\rm{Sel}}}}}}}_{{{{{{{\rm{C}}}}}}}_{{{{{{\rm{n}}}}}}}{{{{{{\rm{H}}}}}}}_{{{{{{\rm{m}}}}}}}}=\frac{{{{{{{\rm{nC}}}}}}}_{{{{{{\rm{n}}}}}}}{{{{{{\rm{H}}}}}}}_{{{{{{{\rm{m}}}}}}}_{{{{{{\rm{outlet}}}}}}}}}{{\sum }_{1}^{{{{{{\rm{n}}}}}}}{{{{{{\rm{nC}}}}}}}_{{{{{{\rm{n}}}}}}}{{{{{{\rm{H}}}}}}}_{{{{{{{\rm{m}}}}}}}_{{{{{{\rm{outlet}}}}}}}}}\times 100 \%$$

Little C_12+_ hydrocarbons were detected. The selectivity to oxygenates was below 1%C and therefore neglected. The carbon balance over the OXZEO catalysts was over 95%.

## Supplementary information

Supplementary Information

Peer Review File

## Data Availability

All data supporting the findings of this study are available within the paper and its supplementary information files.
