# Supplementary material for: Steering the reaction pathway of syngas-to-light olefins with coordination unsaturated sites of ZnGaOx spinel
Source: Nat Commun. 2022 May 18;13:2742. doi: 10.1038/s41467-022-30344-1 (PMC9117195; doi:10.1038/s41467-022-30344-1)
Supplement: Supplementary file 1 — Supplementary Information [file 41467_2022_30344_MOESM1_ESM.pdf]

Supplementary Information

Steering the reaction pathway of syngas-to-light olefins with coordination unsaturated sites of $ZnGaO_x$ spinel

Na Li[1,2,3†], Yifeng Zhu[1,†], Feng Jiao[1,2,3†], Xiulian Pan[1,2,3], Qike Jiang[2], Jun Cai[3,4,5], Yifan Li[1,6], Wei Tong[7], Changqi Xu[2], Shengcheng Qu[2], Bing Bai[1,3], Dengyun Miao[1,2,3], Zhi Liu[4,5], and Xinhe Bao[1,2]**

[1] State Key Laboratory of Catalysis, Dalian Institute of Chemical Physics, Chinese Academy of Sciences, 457 Zhongshan Road, Dalian 116023, (P. R. China)

[2] Dalian National Laboratory for Clean Energy, Dalian Institute of Chemical Physics, Chinese Academy of Sciences, 457 Zhongshan Road, Dalian 116023, (P. R. China)

[3] University of Chinese Academy of Sciences, Beijing 100049, (P. R. China)

[4] State Key Laboratory of Functional Materials for Informatics, Shanghai Institute of Microsystem and Information Technology, Chinese Academy of Sciences, Shanghai 200050, (P. R. China)

[5] School of Physical Science and Technology, ShanghaiTech University, Shanghai 201210, (P. R. China)

[6] Department of Chemical Physics, University of Science and Technology of China, Jinzhai Road 96, Hefei 230026, (P. R. China)

[7] High Magnetic Field Laboratory, Hefei Institutes of Physical Science, Chinese Academy of Sciences, Hefei 230031, (P. R. China)

[†] These authors contributed equally: Na Li, Yifeng Zhu, and Feng Jiao

email: panxl@dicp.ac.cn; xhbao@dicp.ac.cn

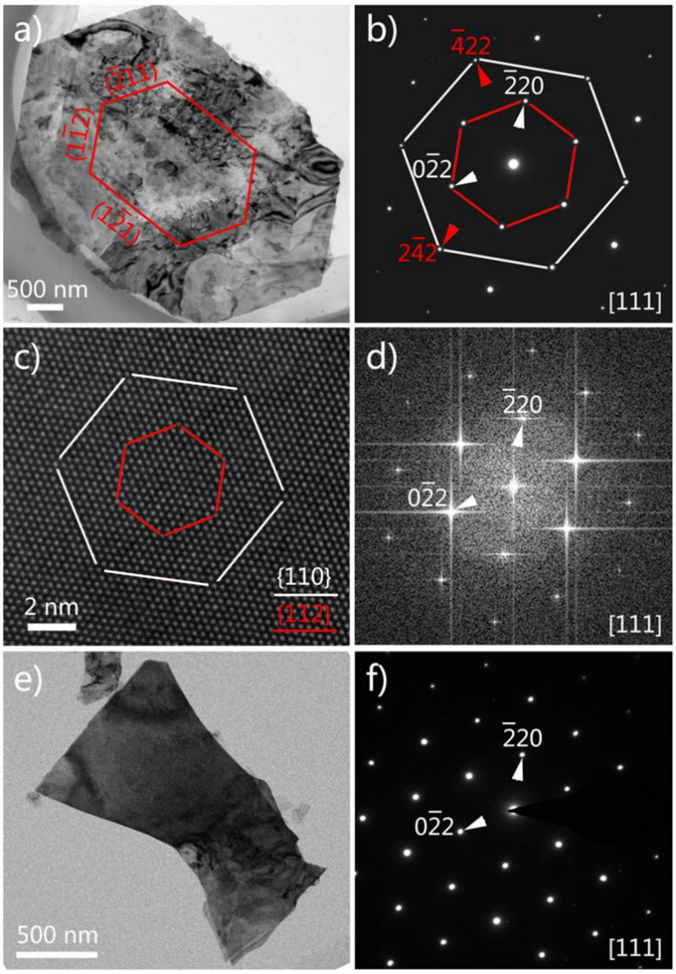

Supplementary Figure 1. **Top view of ZnGaO$_{x_F}$ nanoflakes. a** Low magnification bright field transmission electron microscopy (TEM) image of a hexagonal flake. **b** Corresponding selected area electron diffraction (SAED) pattern. **c** High-resolution transmission electron microscopy (HRTEM) image. **d** Corresponding fast Fourier transform (FFT) pattern. **e** A piece of broken top-view ZnGaO$_{x_F}$ flake. **f** Corresponding SAED pattern. HRTEM images were viewed along the [111] orientation. Note that [uvw] indexed a crystal axis, (hkl) a crystal plane, and {hkl} a group of crystal planes with the same atomic configuration. Numbers of hkl in **b**, **d**, and **f** indicate the crystal planes.

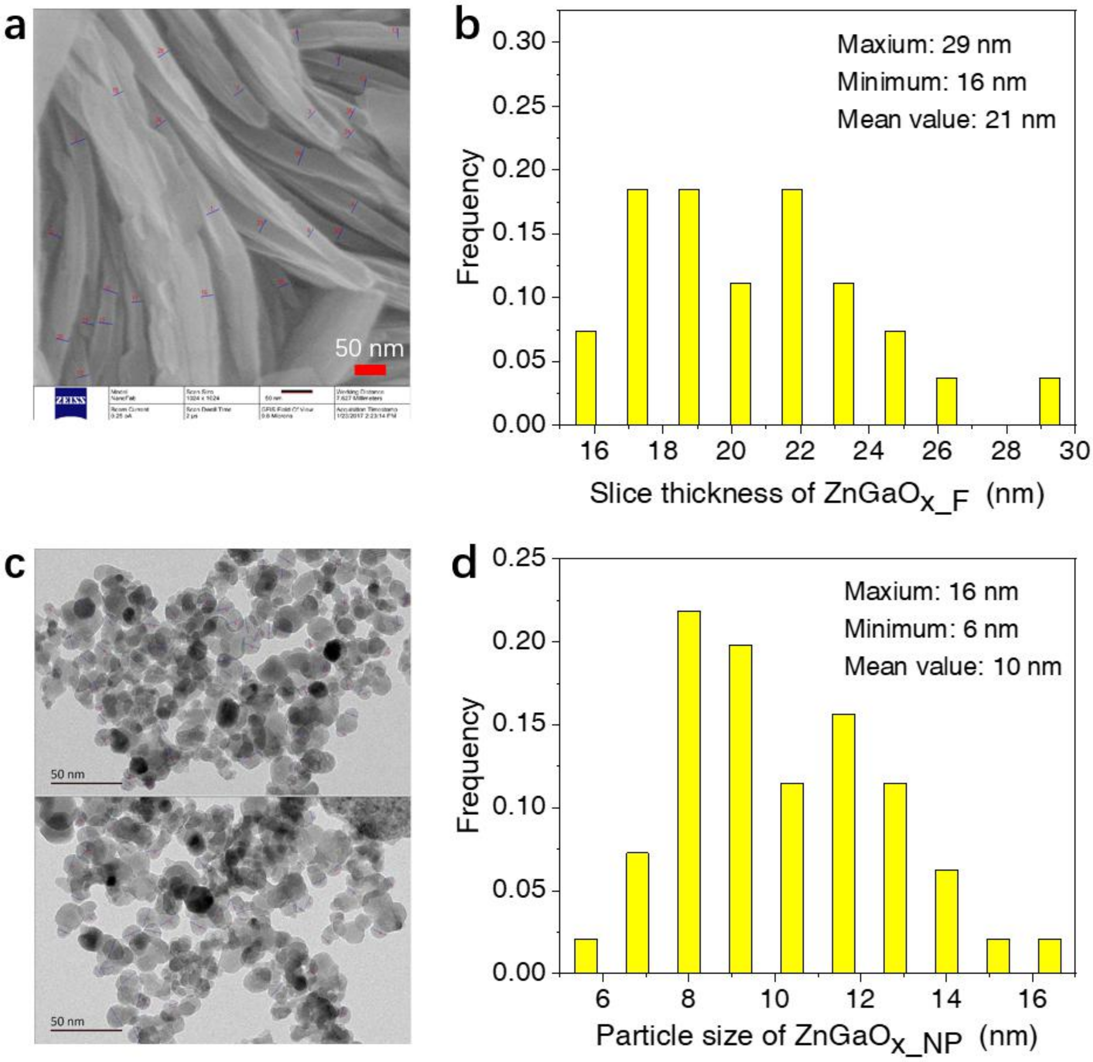

Supplementary Figure 2. **Electron microscopy images and size statistics. a** Scanning electron microscopy (SEM) image of $ZnGaO_{x\_F}$ sample. **b** Corresponding statistical slice thickness. **c** TEM images of $ZnGaO_{x\_NP}$ sample. **d** Corresponding statistical particle size.

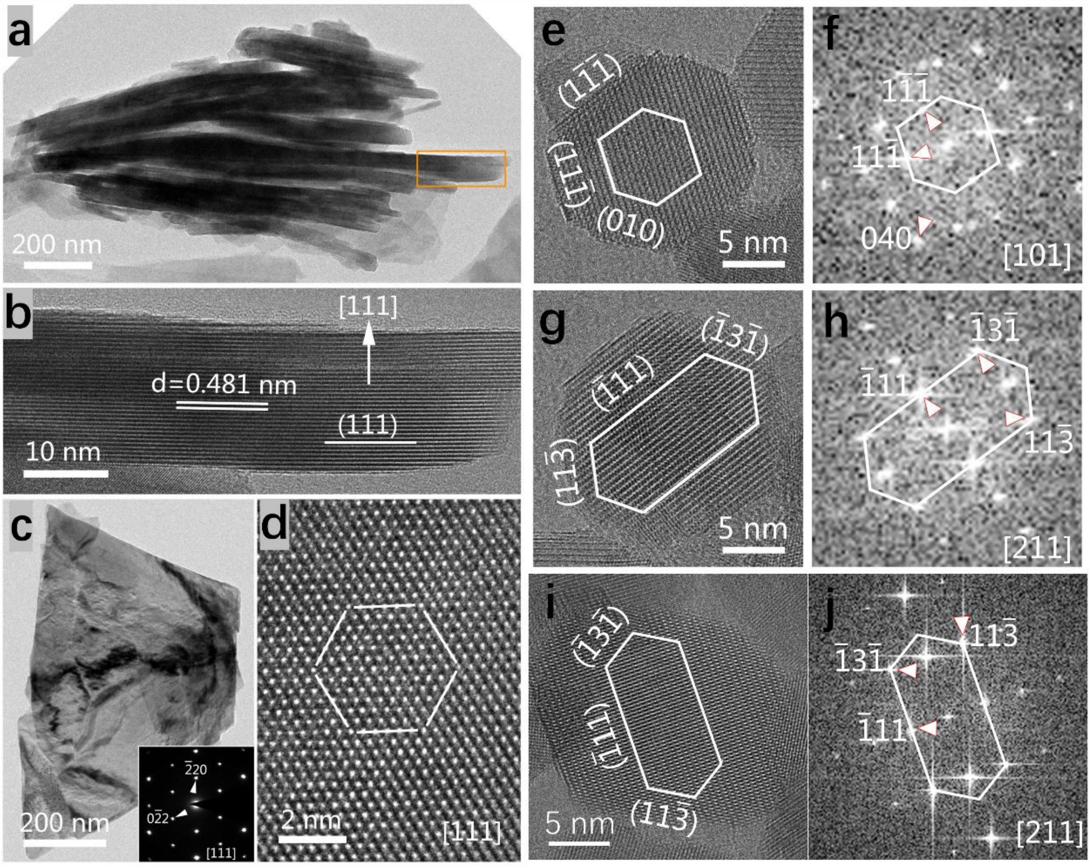

Supplementary Figure 3. **TEM images of used $ZnGaO_x$ samples. a-d** $ZnGaO_{x\_F}$. **e-j** $ZnGaO_{x\_NP}$. **a** Low magnification bright field image of side-view $ZnGaO_{x\_F}$ flake. **b** The enlarged HRTEM image of a selected area with orange frame in **a**. **c** A piece of broken top-view $ZnGaO_x$_F flake. The inset is the corresponding SAED pattern. **d** Corresponding HRTEM image. **e,g,i** Several typical HRTEM images of used $ZnGaO_{x\_NP}$. **f,h,j** Their corresponding FFT patterns. Note that [uvw] indexed a crystal axis, (hkl) a crystal plane, and {hkl} a group of crystal planes with the same atomic configuration. Numbers of hkl in **f**, **h**, **j,** and the inset of **c** indicate the crystal planes.

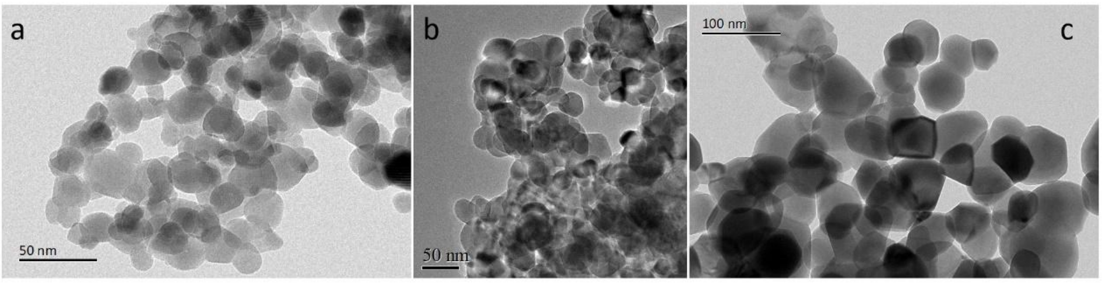

Supplementary Figure 4. **TEM images of $ZnGaO_{x\_NPT}$. a** $ZnGaO_{x\_NP600}$. **b** $ZnGaO_{x\_NP700}$. **c** $ZnGaO_{x\_NP800}$.

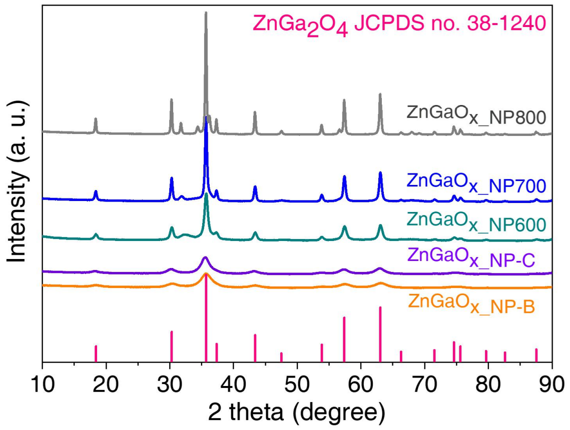

Supplementary Figure 5. **XRD patterns of $ZnGaO_x$ samples.** The diffraction signals of standard $ZnGa_2O_4$ spinel are listed with vertical lines for comparison.

The XRD patterns of $ZnGaO_{x\_NP700}$ and $ZnGaO_{x\_NP800}$ in Supplementary Figure 5 show some diffraction peaks at 2 thetas = ~ 32° and 34° corresponding to (100) and (002) crystal faces of ZnO (JCPDS no. 36-1451) impurity. In order to judge the contributions of this impurity, a $ZnGaO_{x\_NP700}$ sample was chosen and subsequently treated by dilute HNO_3 to remove ZnO impurity ($ZnGaO_{x\_NP700\text{-}leaching}$, XRD in Supplementary Figure 6a). Supplementary Figure 6b shows that the catalytic performance of syngas conversion does not change obviously after the removal of ZnO impurity, which indicates that there is a marginal effect of the few ZnO impurity or the interface of ZnO/$ZnGaO_x$.

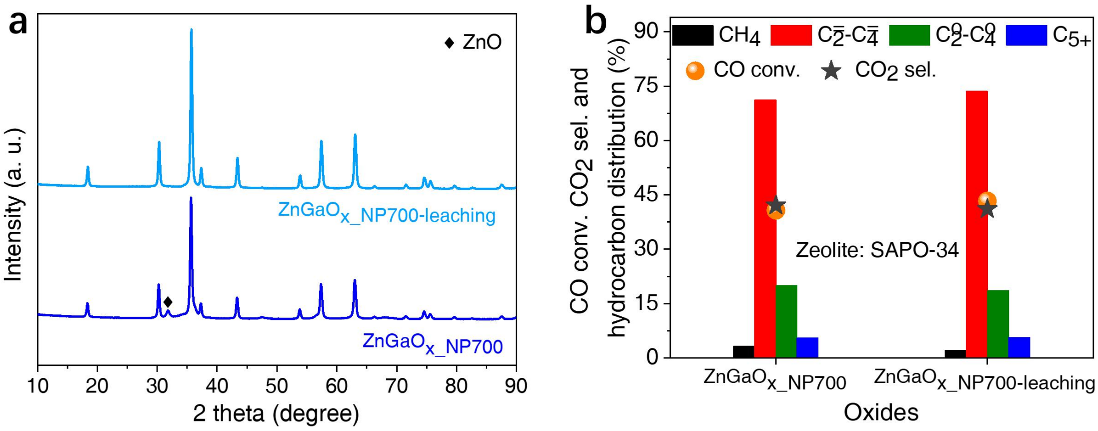

Supplementary Figure 6. **Effect of few ZnO impurity on syngas conversion. a** XRD patterns of $ZnGaO_{x\_NP700}$ and $ZnGaO_{x\_NP700\text{-}leaching}$ samples. **b** Catalytic performances of $ZnGaO_{x\_NP700}$ – SAPO-34 and $ZnGaO_{x\_NP700\text{-}leaching}$ – SAPO-34 catalysts. Reaction conditions: oxide/zeolite mass ratio (OX/ZEO) = 1, H_2/CO = 2.5 (v/v), 400 ºC, 4 MPa, and 1,600 mL g^{-1} h^{-1}.

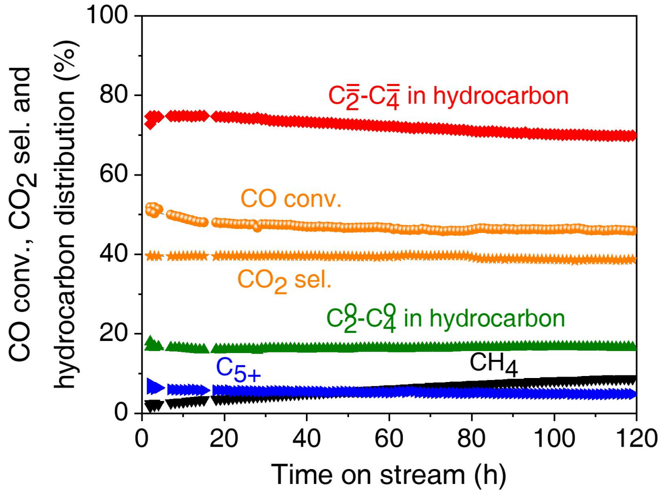

Supplementary Figure 7. **Stability test of ZnGaO$_{x_NP}$−SAPO-34.** A new batch of catalysts was used here. Reaction condition: OX/ZEO = 1, H_2/CO = 2.5 (v/v), 400 ºC, 4 MPa, and 1,600 mL g^{-1} h^{-1}.

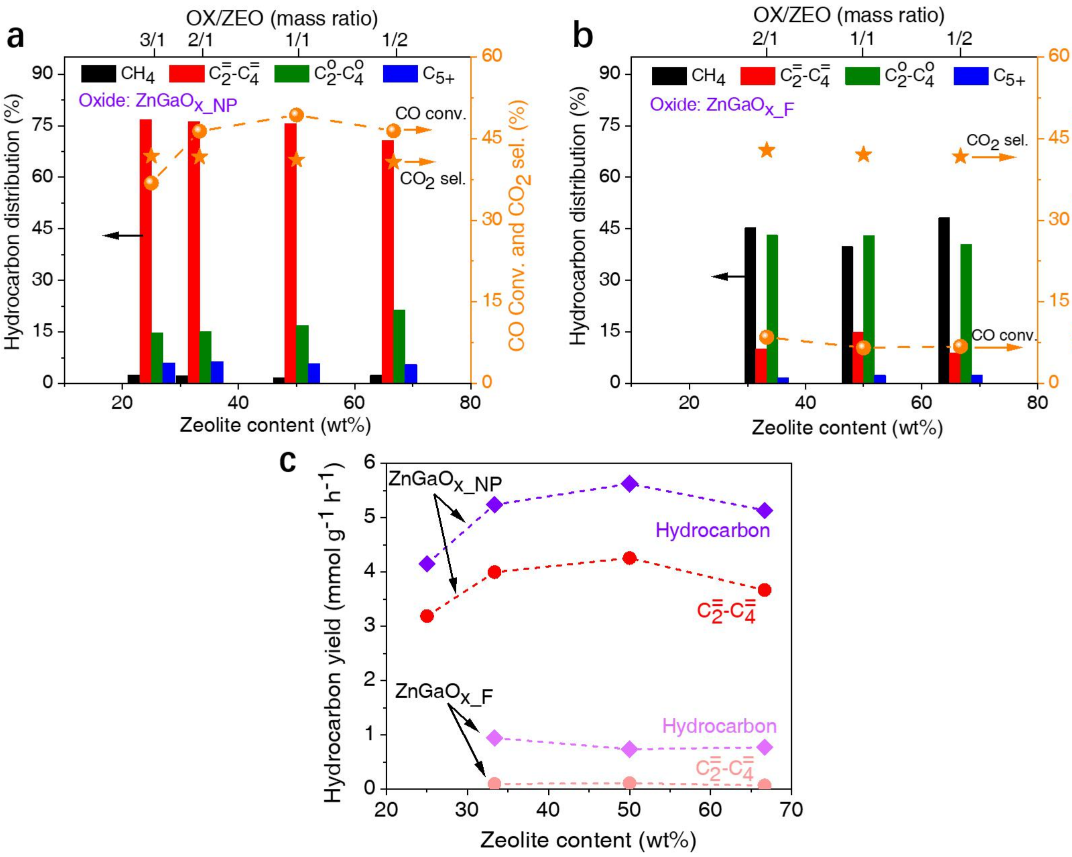

Supplementary Figure 8. **Catalytic performance of $ZnGaO_x$ – SAPO-34 as a function of zeolite content. a** $ZnGaO_{x\_NP}$ – SAPO-34. **b** $ZnGaO_{x\_F}$ – SAPO-34. **c** Comparison of space time yields (STYs) of light olefins ($C_2^=-C_4^=$) and total hydrocarbons as a function of zeolite content. Reaction conditions: $H_2/CO = 2.5$ (v/v), 400 ºC, 4 MPa, 1,600 mL g^{-1} h^{-1}.

It is important to find the appropriate mass ratio of metal oxides to zeolites (OX/ZEO) to ensure the sufficient acid sites of zeolites for the conversion of intermediates generated from oxides, and minimize the limitation of the second step from zeolites in this tandem reaction. We have conducted a series of catalytic performance tests with the mass ratio of metal oxides to zeolites ranging from 3/1 to 1/2 (Supplementary Figure 8a,b), and calculated the corresponding space time yield (STY) of hydrocarbons based on the composites. As displayed in Supplementary Figure 8c, the STYs of hydrocarbons (light olefins and hydrocarbon) vary obviously for $ZnGaO_{x\_NP}$ with the highest value achieved at the ratio of 1/1 (50 wt% zeolite content). Further enhancing zeolite content, STYs of hydrocarbons will not increase, which indicates the oxide is insufficient above this ratio. Similarly, the decrease in STYs below 50 wt% content is due to the insufficient content of zeolites below this ratio. Therefore, 50 wt% zeolite is enough to ensure a high activity and meanwhile reduce the limitation of zeolite for $ZnGaO_{x\_NP}$. In contrast, STYs remain almost unchanged for $ZnGaO_{x\_F}$, which indicates that the intermediates generated from $ZnGaO_{x\_F}$ cannot be effectively converted to desired products by SAPO-34 catalysis, and thus there is no limitation from the second step in zeolites for $ZnGaO_{x\_F}$.

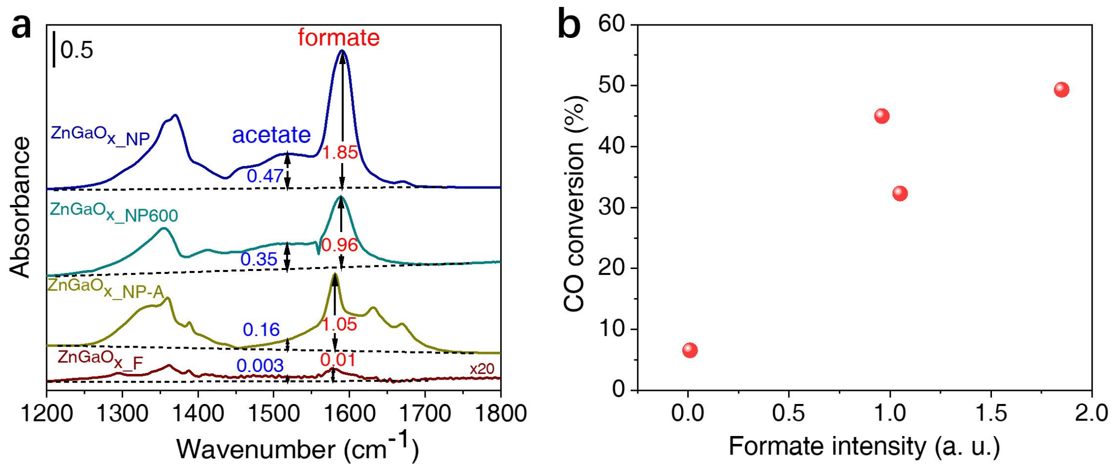

Supplementary Figure 9. *In-situ* FT-IR differential spectra of syngas conversion over H_2-reduced $ZnGaO_x$ samples. **a** Samples of $ZnGaO_{x\_NP}$, $ZnGaO_{x\_NP600}$, $ZnGaO_{x\_NP\text{-}A}$, and $ZnGaO_{x\_F}$. **b** Relationship between formate intensity around 1589 cm^{-1} and CO conversion.

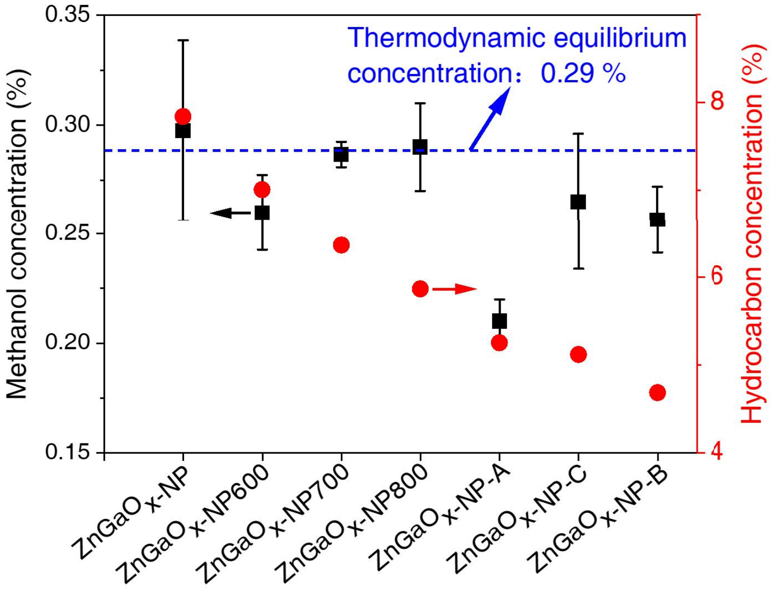

Supplementary Figure 10. Relationship between methanol concentration and hydrocarbon formation. Methanol formation activity over $ZnGaO_x$ oxides alone and hydrocarbon formation activity over $ZnGaO_{x\_NP}$–SAPO-34 bifunctional catalysts were compared. Methanol concentration in outlet gas were tested 3-5 batches for each $ZnGaO_x$ catalyst. Error bars represent standard deviation, n = 3-5 independent replicates. Black square indicates mean value. Reaction conditions: H_2/CO = 2.5 (v/v), 400 ºC, 4 MPa, 3,200 mL g^{-1} h^{-1}.

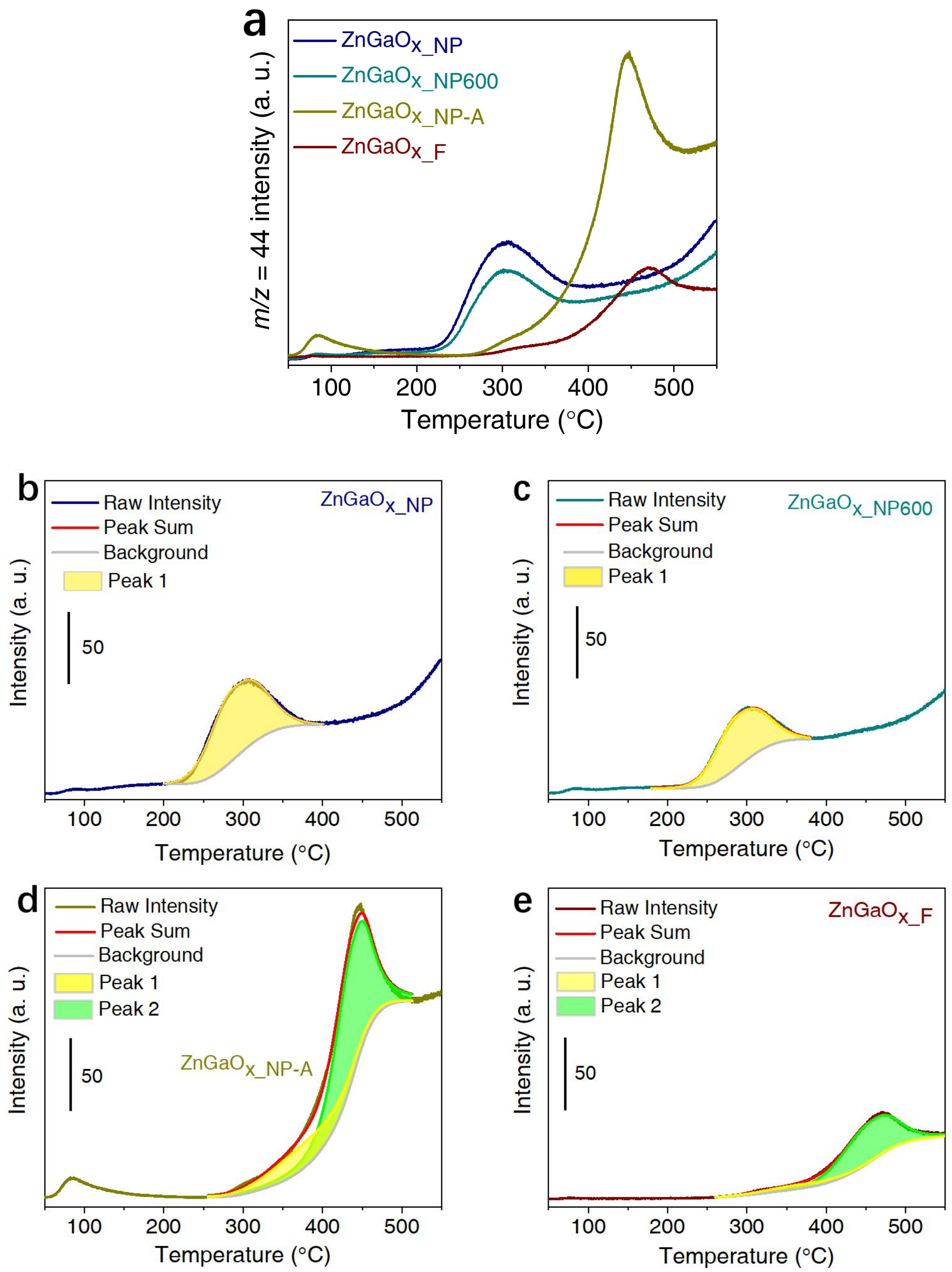

Supplementary Figure 11. **CO-TPR and its fitting curves. a** $ZnGaO_x$ samples. **b** $ZnGaO_{x\_NP}$. **c** $ZnGaO_{x\_NP600}$. **d** $ZnGaO_{x\_NP-A}$. **e** $ZnGaO_{x\_F}$. The integral area of Peak 1 represented the amount of CO_2 produced below reaction temperature of 400 ºC, and that of peak 2 represented the amount of CO_2 produced above 400 ºC. Supplementary Table 4 listed the detailed integration parameters. Red and greyish lines refer to peak sum and background separately in the fitting curves.

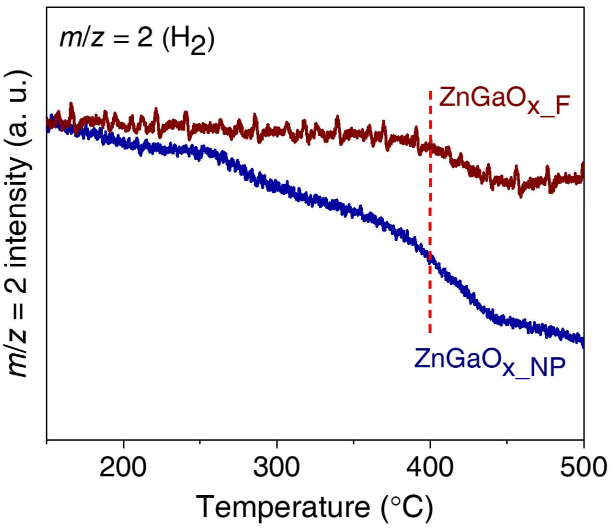

Supplementary Figure 12. H_2-TPR profiles of $ZnGaO_x$ samples. *m*/*z* = 2 (H_2) signals in the effluents were monitored by an online mass spectrometer. The red vertical dash line corresponds to 400 ºC.

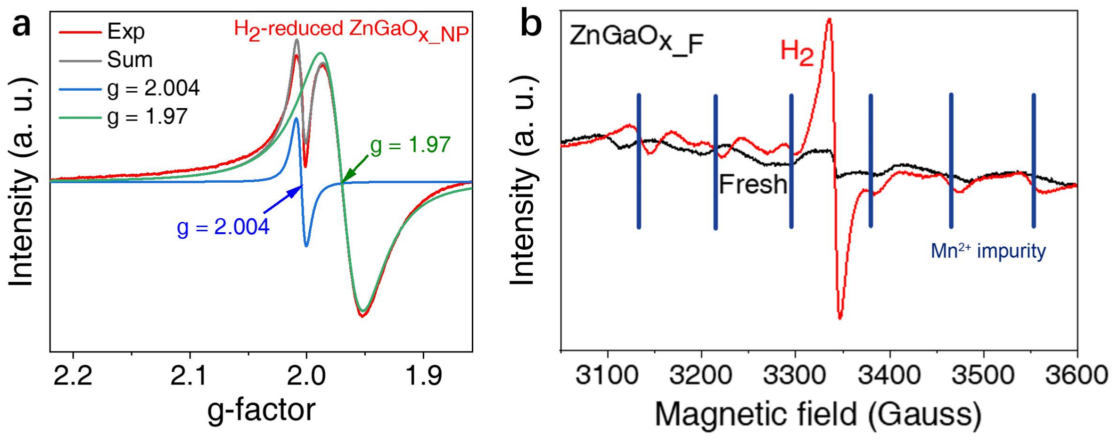

Supplementary Figure 13. Fitting curves of *quasi-in-situ* EPR spectra. a H_2-reduced $ZnGaO_{x\_NP}$. The red, gray, blue, and green lines refer to experimental, peak sum, g = 2.004, and g = 1.97 signals, respectively. **b** EPR spectra of the $ZnGaO_{x\_F}$ in Fig. 4d, and the six blue vertical lines indicate the signal of Mn^{2+} impurity.

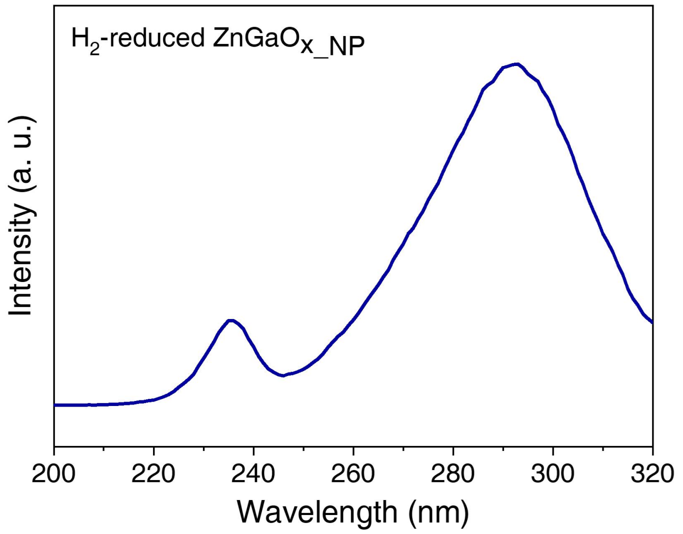

Supplementary Figure 14. **Excitation spectrum of H_2-reduced $ZnGaO_{x\_NP}$.** The emission wavelength is fixed at 345 nm.

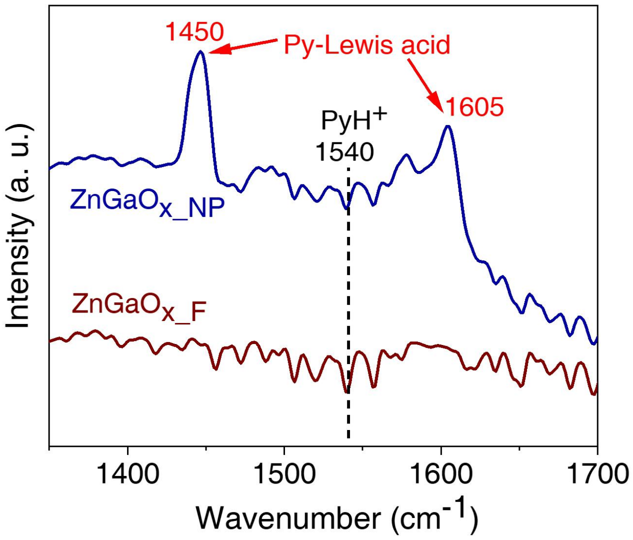

Supplementary Figure 15. *In-situ* FT-IR differential spectra of pyridine adsorption. Samples are $ZnGaO_{x\_NP}$ (navy line) and $ZnGaO_{x\_F}$ (wine line), respectively. Signals at 1450 and 1605 cm^{-1} are associated with pyridine adsorbed on Lewis acid sites (Py-Lewis acid), and that at 1540 cm^{-1} corresponds to Brønsted acid sites (PyH^+).

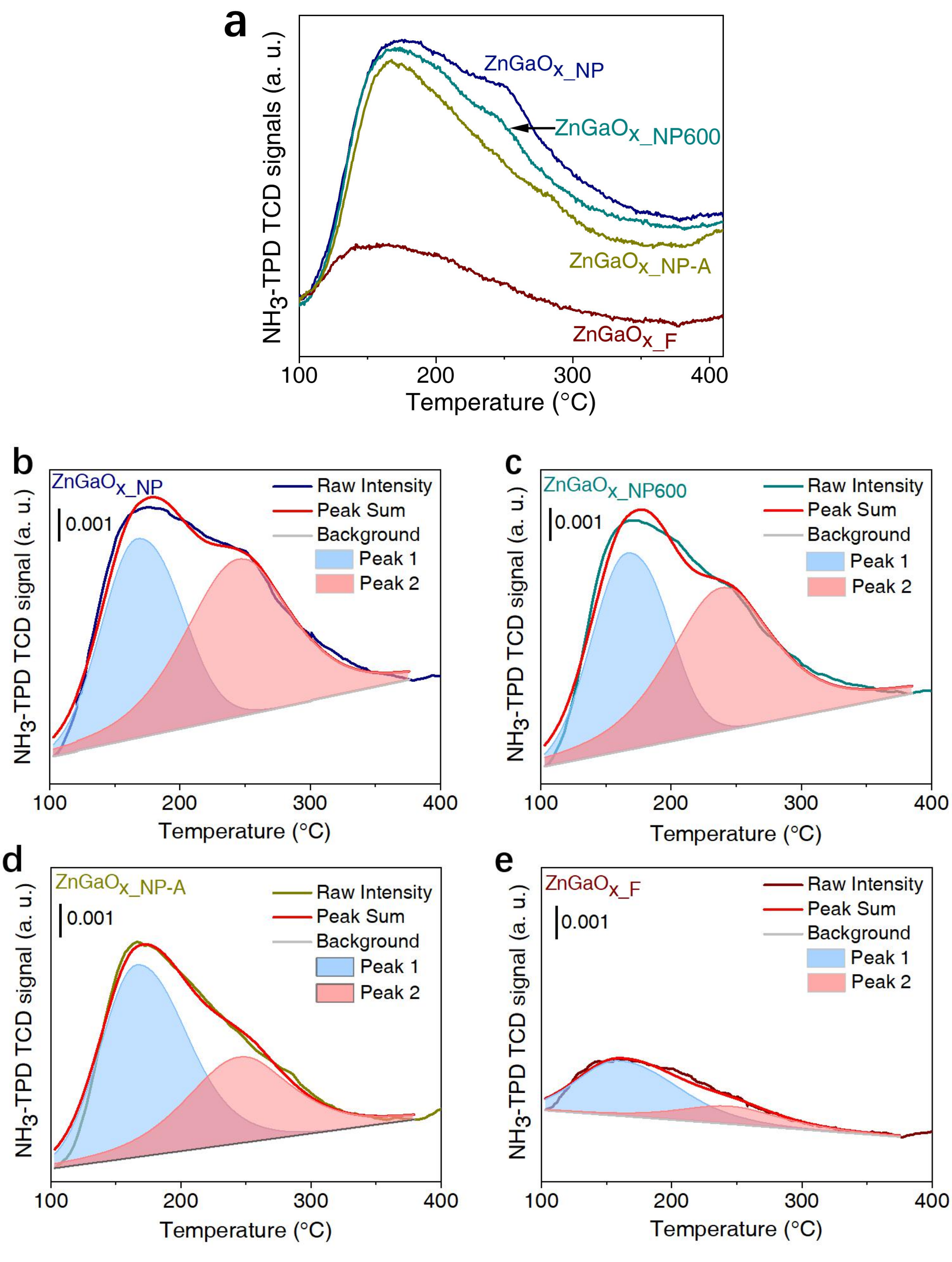

Supplementary Figure 16. **NH_3-TPD and its fitting curves. a** $ZnGaO_x$ samples. **b** $ZnGaO_{x\_NP}$. **c** $ZnGaO_{x\_NP600}$. **d** $ZnGaO_{x\_NP-A}$. **e** $ZnGaO_{x\_F}$. The integral area of Peak 1 represented the amount of weak strength acid sites, and that of peak 2 represented the amount of medium strength acid sites. Supplementary Table 8 listed the detailed integration parameters.

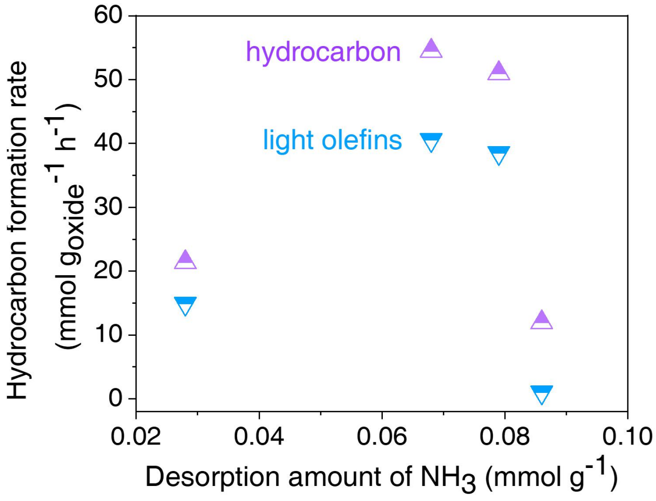

Supplementary Figure 17. **Hydrocarbon formation rate as a function of the amount of weak strength acid sites of ZnGaO$_x$ samples**. Samples from left to right are ZnGaO$_{x_NP\text{-}A}$, ZnGaO$_{x_NP600}$, ZnGaO$_{x_NP}$, and ZnGaO$_{x_F}$.

Supplementary Table 1. Relative elemental composition, crystal size, and pore structure of oxidic $ZnGaO_x$ samples.

Oxide	Zn/Ga molar ratio		Mn impurity (ppm)[a]	Average crystal size (nm)[d]	Specific surface area (m^2 g^{-1})	Total pore volume (cm^3 g^{-1})	The most probable pore width (nm)
	Bulk[a,b]	Surface[c]					
$ZnGaO_{x\_NP}$	0.83[a]	1.2	2.1	11	60	0.23	16.7
$ZnGaO_{x\_F}$	0.24[a,b]	0.2	5.0	-	31	0.07	5.3
$ZnGaO_{x\_NP600}$	0.71[b]	1.4	-	21	36	0.20	16.7
$ZnGaO_{x\_NP700}$	0.71[b]	1.2	-	39	20	0.13	24.6
$ZnGaO_{x\_NP800}$	-	3.1	-	67	13	0.05	31.5
$ZnGaO_{x\_NP\text{-}A}$	0.16[a]	0.2	-	3	125	0.14	4
$ZnGaO_{x\_NP\text{-}B}$	0.28[a]	0.3	-	4	116	0.15	5
$ZnGaO_{x\_NP\text{-}C}$	0.39[a]	0.4	-	6	98	0.19	6.5

Determined by [a] inductively coupled plasma optical emission spectrometer (ICP-OES), [b] Scanning electron microscopy with energy dispersive X-ray detector (SEM-EDX), and [c] X-ray photoelectron spectroscopy (XPS) results of fresh oxidic samples. [d] $ZnGaO_x$ crystal sizes were calculated using the Scherrer equation. “-” represented not measured.

Supplementary Table 2. Reaction performance of syngas conversion over $ZnGaO_x$–SAPO-34.[a]

Oxide	CO conv. (%)	CO_2 sel. (%)	Methanol sel. (%)	Hydrocarbon distribution (%)				C_2-C_4 O/P[b]
				CH_4	$C_2^=$-$C_4^=$	C_2^o-C_4^o	C_{5+}	
$ZnGaO_{x\_NP}$	49.3	41.1	0.06	1.7	75.6	16.9	5.8	4.5
$ZnGaO_{x\_F}$	6.6	42.0	0.51	39.8	14.9	42.9	2.4	0.3
$ZnGaO_{x\_NP600}$	45.0	42.3	0.04	1.9	75.8	16.7	5.6	4.6
$ZnGaO_{x\_NP700}$	40.7	42.1	0.03	3.3	71.2	20.0	5.5	3.6
$ZnGaO_{x\_NP800}$	37.0	41.3	0.07	3.8	69.0	21.5	5.6	3.2
$ZnGaO_{x\_NP-A}$	32.3	39.8	1.4	1.6	77.6	15.8	5.0	4.9
$ZnGaO_{x\_NP-B}$	29.0	40.2	1.7	2.3	72.5	19.9	5.3	3.6
$ZnGaO_{x\_NP-C}$	31.8	40.4	2.1	1.9	78.5	14.1	5.5	5.6

[a] Reaction conditions: OX/ZEO = 1 (mass ratio), H_2/CO = 2.5 (v/v), 400 ºC, 4.0 MPa, GHSV = 1,600 mL g^{-1} h^{-1}. [b] O/P represented the selectivity ratio of light olefins ($C_2^=$-$C_4^=$) to light paraffins (C_2^o-C_4^o).

Supplementary Table 3. Reaction performance of syngas conversion over individual $ZnGaO_x$ catalysts.[a]

Catalyst	CO conv. (%)	Product selectivity (%)[b]			Hydrocarbon distribution (%)			
		CO_2	Methanol	Hydrocarbon	CH_4	$C_2^{=}$-$C_4^{=}$	C_2^{o}-C_4^{o}	C_{5+}
$ZnGaO_{x\_NP}$	5.5	40.8	23.9	35.3	68.6	13.9	15.8	1.7
$ZnGaO_{x\_F}$	5.8	43.2	4.8	52.0	97.3	0.5	1.9	0.3

[a] Reaction conditions: H_2/CO = 2.5 (v/v), 400 ºC, 4 MPa, 3,200 mL g^{-1} h^{-1}. [b] Product selectivity here was calculated using normalized data on the carbon basis. Methanol equilibrium concentration is 0.29% at current reaction conditions. Methanol concentration in outlet gas of all oxides samples was displayed in Supplementary Figure 10 for comparison.

Supplementary Table 4. The detailed fitting parameters of CO-TPR profiles in Supplementary Figure 11.[a]

Sample	BG type and range (ºC)	Peak 1					
		Position (ºC)	FWHM (ºC)	M	TS	TL	Area (a. u.)
$ZnGaO_{x\_NP}$	Shirley 200-402	287	65	0	0.8	18	4860
$ZnGaO_{x\_NP600}$	Shirley 180-380	287	61	0	0.8	18	3298
$ZnGaO_{x\_NP-A}$	Shirley 256-513	381	108	0	1	0.01	1963
$ZnGaO_{x\_F}$	Shirley 260-570	369	107	0	1	0.1	328
		Peak 2					
$ZnGaO_{x\_NP}$	Shirley 200-402	>400	--	--	--	--	0
$ZnGaO_{x\_NP600}$	Shirley 180-380	>400	--	--	--	--	0
$ZnGaO_{x\_NP-A}$	Shirley 256-513	438	51	45	1	3	6524
$ZnGaO_{x\_F}$	Shirley 260-570	454	77	16	1	3	2576

[a] Note: M = Gaussian-Lorentzian mixing (0 Gaussian: 1 Lorentzian), TS = asymmetry parameter, TL = asymmetry tailing parameter. FWHM: full width at half maximum. BG: background.

Supplementary Table 5. Integral area of CO_2 peak in the CO-TPR profiles of $ZnGaO_x$ samples.

Oxide	Integral area of CO_2 peak (a. u.)	
	<400 ºC[a]	>400 ºC[a]
$ZnGaO_{x\_NP}$	4860	0
$ZnGaO_{x\_NP600}$	3341	0
$ZnGaO_{x\_NP-A}$	1963	6524
$ZnGaO_{x\_F}$	328	2576

[a] Range of peak temperature.

Supplementary Table 6. Surface composition of $ZnGaO_x$ analyzed by AP-XPS.

Oxide	Treatment condition	Surface element content (%)			Zn/Ga molar ratio	O/Ga molar ratio	O/Zn molar ratio
		Zn	Ga	O			
$ZnGaO_{x\_NP}$	UHV-O_2	19	26	56	0.73	2.15	2.95
	H_2	11	30	59	0.37	1.97	5.36
$ZnGaO_{x\_F}$	UHV-O_2	7	35	58	0.20	1.66	8.29
	H_2	5	34	60	0.15	1.76	12.0

Note: UHV: ultra-high vacuum.

Supplementary Table 7. NH_3-TPD analysis of $ZnGaO_x$ samples.

Oxide	Desorption amount of NH_3 (mmol g^{-1})		
	Weak	Medium	Total
$ZnGaO_{x\_NP}$	0.079	0.091	0.17
$ZnGaO_{x\_NP600}$	0.068	0.082	0.15
$ZnGaO_{x\_NP-A}$	0.028	0.052	0.13
$ZnGaO_{x\_F}$	0.086	0.009	0.04

Supplementary Table 8. The detailed fitting parameters of NH_3-TPD profiles in Supplementary Figure 16.[a]

Sample	BG type and range (ºC)	Peak 1: weak acid					
		Position (ºC)	FWHM (ºC)	M	TS	TL	Acid density (mmol g^{-1})
$ZnGaO_{x\_NP}$	Linear 103-377	167	64	0	0.5	15	0.078
$ZnGaO_{x\_NP600}$	Linear 103-384	165	63	0	0.5	10	0.069
$ZnGaO_{x\_NP\text{-}A}$	Linear 104-380	166	65	0	0.5	30	0.028
$ZnGaO_{x\_F}$	Linear 103-375	160	75	0	0.5	30	0.086
		Peak 2: medium strength acid					
$ZnGaO_{x\_NP}$	Linear 103-377	244	95	43	0.5	0.6	0.091
$ZnGaO_{x\_NP600}$	Linear 103-384	238	94	55	0.5	0.6	0.082
$ZnGaO_{x\_NP\text{-}A}$	Linear 104-380	244	95	50	0.5	0.6	0.052
$ZnGaO_{x\_F}$	Linear 103-375	244	90	50	0.5	0.6	0.009

[a] Note: M = Gaussian-Lorentzian mixing (0 Gaussian: 1 Lorentzian), TS = asymmetry parameter, TL = asymmetry tailing parameter.
